# Metabolomic Profiling of Pregnancies With Polycystic Ovary Syndrome Identifies a Unique Metabolic Signature and Potential Predictive Biomarkers of Low Birth Weight

**DOI:** 10.3389/fendo.2021.638727

**Published:** 2021-06-15

**Authors:** Ilhame Diboun, Manjunath Ramanjaneya, Lina Ahmed, Mohammed Bashir, Alexandra E. Butler, Omar Albagha, Abdul Badi Abou-Samra, Stephen L. Atkin, Nayef A. Mazloum, Mohamed A. Elrayess

**Affiliations:** ^1^ College of Health and Life Sciences, Hamad Bin Khalifa University (HBKU), Doha, Qatar; ^2^ Qatar Metabolic Institute, Hamad Medical Corporation, Doha, Qatar; ^3^ Translational Research Institute, Hamad Medical Corporation, Doha, Qatar; ^4^ Department of Microbiology and Immunology, Weill Cornell Medicine-Qatar, Qatar Foundation, Doha, Qatar; ^5^ Diabetes Research Center (DRC), Qatar Biomedical Research Institute (QBRI), Hamad Bin Khalifa University (HBKU), Qatar Foundation (QF), Doha, Qatar; ^6^ Centre for Genomic and Experimental Medicine, Institute of Genetics and Molecular Medicine, University of Edinburgh, Edinburgh, United Kingdom; ^7^ Post Graduate Studies and Research, Royal College of Surgeons in Ireland Bahrain, Adliya, Bahrain; ^8^ Biomedical Research Center (BRC), Qatar University, Doha, Qatar

**Keywords:** metabolomics, pregnancy, polycystic ovary syndrome (PCOS), birth weight, arachidonic acid, linoleic acid, palmitic acid

## Abstract

**Background:**

Polycystic ovary syndrome (PCOS) is a complex syndrome with clinical features of an endocrine/metabolic disorder. Various metabolites show significant association with PCOS; however, studies comparing the metabolic profile of pregnant women with and without PCOS are lacking. In this study, metabolomics analysis of blood samples collected from PCOS women and age and BMI matched controls in the second trimester of pregnancy was performed to identify metabolic differences between the two groups and determine their association with pregnancy outcome.

**Methods:**

Sixteen PCOS and fifty-two healthy women in their second trimester underwent targeted metabolomics of plasma samples using tandem mass spectrometry with the Biocrates MxP^®^ Quant 500 Kit. Linear regression models were used to identify the metabolic alterations associated with PCOS, followed by enrichment and Receiver Operating Characteristic (ROC) analyses to determine the best indicators of pregnancy outcomes.

**Results:**

PCOS women had lower birth weight babies compared to healthy controls. As a group, systolic blood pressure (SBP) at both second trimester and at delivery negatively correlated with birth weight. Regression models indicated significant increases in the triglycerides C20:4_C34:3 and C18:2_C38:6 in the PCOS group [false discovery rate (FDR) <0.05]. Enrichment analysis revealed significant elevations in triglycerides containing arachidonic acid, linoleic acid and palmitic acid in the PCOS group. A number of indicators of baby birth weight were identified including SBP at delivery, hexosylceramide (d18:2/24:0), ceramide (d18.0/24.1) and serine, with an AUC for all predictors combined for low birth weight (≤2500grams) of 0.88 (95%CI: 0.75-1.005, p<0.001).

**Conclusions:**

PCOS pregnancies resulted in babies with a lower birth weight, marked by a unique metabolic signature that was enriched with specific triglycerides and unsaturated fatty acids. The functional significance of these associations needs further investigation.

## Introduction

Pregnancy triggers significant metabolic alterations to meet the increasing physiological demands of the mother and her developing fetus (1). In early pregnancy, these alterations reflect the whole body’s anabolic status to fulfill the increasing nutritional needs; however, these physiological metabolic changes may be greatly influenced by various underlying health conditions (2). Among these, polycystic ovary syndrome (PCOS) affecting up to 10% of reproductively active women (3), causes anovulatory infertility (4) and increased risk of adverse pregnancy outcomes, including gestational diabetes, miscarriage and low birth weight (5, 6). Metabolic changes associated with PCOS in pregnant women and their impact on birth weight have not been fully explored.

Significant advances in mass spectrometry technologies have revolutionized the discovery of metabolic pathways that underlie the progression of various diseases such as insulin resistance, type 2 diabetes (7–9), cancer (10) and cardiovascular disease (11), giving a deeper insight into their disease etiologies. Studies have investigated changes in metabolites in different biofluids and tissues and have identified various potential markers with significant diagnostic and therapeutic utility (12). Studies of reproductive diseases, such as PCOS, GDM and poor pregnancy outcomes, have received increasing interest (13–15). A recent study investigating the metabolic changes associated with PCOS in pregnant women indicated abnormalities in lipid metabolism and beta-oxidation of fatty acids, causing increased risk of miscarriage (16). However, metabolic profiling to predict risk of low birth weight deliveries has not yet been explored.

In this study, targeted metabolomics analysis of blood samples from age and weight matched pregnant women in their second trimester with and without PCOS was related to pregnancy outcomes, including birth weight and gestational age at delivery. The emerging data indicate a unique metabolic signature of PCOS pregnant women at second trimester and identify metabolic markers of low birth weight.

## Methods

### Study Design

This is a cross-sectional study that included 68 pregnant women (52 controls and 16 with PCOS) recruited during their second trimester at the antenatal clinic at The Women Wellness and Research Center (WWRC) of Hamad Medical Corporation in Doha, Qatar. The diagnosis of PCOS was based on the NIH criteria of biochemical evidence of hyperandrogenemia (free androgen index >4.5) or a raised testosterone greater than 2.7nmol/l, and oligomenorrhea or amenorrhea. Transvaginal ultrasound was not culturally acceptable and the abdominal ultrasounds in this obese population inaccurate enough to confirm polycystic ovaries. Non-classical 21-hydroxylase deficiency, hyperprolactinemia, Cushing’s disease and androgen‐secreting tumors were excluded by appropriate tests. All subjects had an oral glucose tolerance test to exclude diabetes. Protocols were approved by Institutional Review Boards (IRBs) of the Hamad Medical Corporation (15101/15) and Weill Cornell Medicine in Qatar (15-00016). All patients gave their written informed consent and the study was conducted in accordance with ICH GCP and the Declaration of Helsinki. Anthropometrics, medical history and demographics data were collected. Blood samples were collected by venipuncture during the regular follow-up second trimester visits and transported to the main HMC laboratory for the routine patient tests including biochemical profile. Additional samples serum were stored at -80°C until analysis was undertaken. Laboratory tests included second trimester biochemical profile and thyroid function tests. Blood samples were also collected for metabolomics analysis. Pregnancy outcomes including gestational age at delivery, birth weight, maternal weight and blood pressure were collated with the metabolomic profile for all participants.

### Metabolomics

Tandem mass spectrometry was performed for targeted metabolomics of plasma samples using Biocrates MxP^®^ Quant 500 Kit (Biocrates, Innsbruck, Austria) at the Fraunhofer Institute for Toxicology and Experimental Medicine (ITEM). FIA-MS/MS was utilized to measure lipids using a 5500 QTRAP^®^ instrument with an ESI source (AB Sciex, Darmstadt, Germany). LC-MS/MS utilizing the same 5500 QTRAP^®^ instrument was used for measuring small molecules as described previously (17). Appropriate MS software (Sciex Analyst^®^) was used to quantify data that was then imported into Biocrates MetIDQ™ software for calculation of analyte concentrations, assessment and compilation of data.

### Statistical Analysis

Statistical analyses of demographic traits were performed using IBM SPSS version 25, R version 3.2.1 and SIMCA 14.0 software (Umetrics, Sweden). Variables with skewed distributions were log transformed to ensure normality (18). Differences between controls and PCOS were tested by independent sample t test (normally distributed variables) or Mann-Whitney U (variables with skewed distribution) test. A p-value significance level of 0.05 was used. Data are presented as mean (SD) in tables or median (interquartile range) in figures. For metabolomics data analysis, principle component analysis (PCA) was performed using R version 2.14, www.r-project.org/. PCA revealed two main components (PC1 and PC2) that together captured 24% of the variance in the data (not shown). Orthogonal partial least square discriminant analysis (OPLS-DA), implemented as part of the software SIMCA, was used to compare controls and PCOS groups. All metabolites with greater than 50% missing values were omitted from SIMCA analysis. Linear regression was performed to identify significant metabolites differentiating PCOS from matching controls using the R statistical package (version 2.14, www.r-project.org/) after correcting for age, BMI and principle components (PC1 and PC2). Function enrichment analysis was performed using Chi square test by considering metabolites with a nominal p-value less than 0.05 from linear regression analysis by assessing the probability of observing the associated nominally-significant metabolites from the linear model by pure chance. The biological categories tested for enrichment were provided by Biocrates and expanded manually by reference to the Human Metabolome Database (19). An automated linear regression model, followed by Receiver Operating Characteristic (ROC) analysis, was utilized to determine the best predictors of pregnancy outcomes using IBM SPSS version 25.

## Results

### General Characteristics of Participants

**Figure 1:**
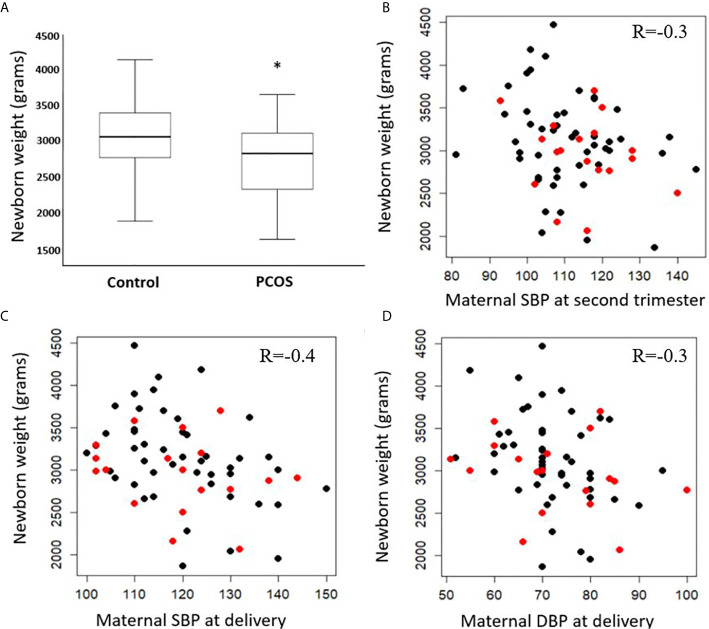
Difference in birth weight at delivery in control and PCOS groups **(A)** median, interquartile range and maximum and minimum values). Correlations between birth weight (PCOS in red, controls in black) and systolic blood pressure (SBP) of mothers at second trimester **(B)**, SBP at delivery **(C)**, and diastolic blood pressure (DBP) at delivery **(D)**. Significance level (*) ≤ 0.05.

PCOS women in their second trimester of pregnancy were insulin resistant accordingly to their homeostatic model assessment for insulin resistance (HOMA-IR), and had on average 339.3 gram lighter babies compared to gestational-age and weight matched healthy controls on delivery (p<0.05) (**Table 1** and **Figure 1A**). The birth weights correlated negatively with overall maternal SBP at second trimester (R=-0.3, p=0.01) (**Figure 1B**) as well as SBP (R=-0.4, p=0.003) and DBP (R=-0.3, p=0.03) at delivery (**Figures 1C, D** respectively). As expected, there was a significant positive correlation between gestational age at delivery and birth weight (R=0.4, p<0.001). There were no significant differences in other pregnancy outcomes including the gestational age at delivery.

**Table 1:** General characteristics of study participants.

Time	Variables	Total	Controls	PCOS	P valueControl *vs* PCOS
		**(N=68)**	**(N=52)**	**(N=16)**
**Second Trimester**	**Age (years)**	31.8 (5.7)	31.2 (5.8)	33.9 (4.9)	0.09
	**BMI (kg/m2)**	32.2 (7.1)	31.6 (7.2)	34.3 (6.5)	0.18
	**SBP (mmHg)**	111.9 (12.5)	111.2 (12.7)	114.0 (12.0)	0.44
	**DBP (mmHg)**	63.8 (7.7)	63.6 (7.6)	64.4 (8.3)	0.70
	**Cholesterol (mmol.L)**	4.8 (1.1)	4.8 (1.1)	4.8 (0.9)	0.99
	**Triglycerides (mmol.L)**	1.5 (0.8)	1.4 (0.7)	1.7 (0.9)	0.36
	**HDL (mmol.L)**	1.3 (0.4)	1.3 (0.40)	1.3 (0.6)	0.92
	**LDL (mmol.L)**	2.8 (0.9)	2.9 (0.9)	2.8 (1.0)	0.86
	**Insulin (uIU.L)**	0.5 (0.4)	0.4 (0.3)	0.6 (0.4)	0.06
	**HbA1c(%)**	5.3 (0.5)	5.3 (0.5)	5.3 (0.5)	0.87
	**HOMA-IR**	1.8 (1.7)	1.5 (1.7)	2.7 (1.3)	0.04
	**Vitamin D_3_ (IU.L)**	15.1 (5.9)	14.9 (6.1)	15.9 (5.6)	0.60
	**Urea (nmol.L)**	3.1 (1.2)	3.1 (1.1)	2.9 (1.2)	0.43
	**Creatinine (nmol.L)**	49.7 (9.1)	49.4 (9.7)	51.7 (6.7)	0.39
	**ALT(mmol.l)**	15.1 (10.0)	13.9 (8.6)	18.9 (12.8)	0.08
	**AST(mmol.l)**	17.1 (7.1)	16.7 (6.9)	16.5 (7.8)	0.91
	**Bilirubin (umol.L)**	7.5 (4.4)	7.3 (3.9)	8.1 (5.6)	0.54
	**ALP (iu.L)**	83.3 (35.2)	84.3 (33.8)	80.3 (40.2)	0.70
	**Albumin (g.L)**	34.6 (6.7)	34.9 (7.0)	33.8 (5.9)	0.56
	**TSH (mU.L)**	2.4 (3.4)	2.7 (3.8)	1.6 (0.7)	0.29
	**Thyroxine (pmol.L)**	12.5 (1.8)	12.4 (1.6)	12.9 (2.3)	0.32
	**Total Protein (g.L)**	66.3 (5.3)	66.3 (5.6)	66.2 (4.8)	0.96
	**Gestational age (weeks)**	21.0 (4.4)	21.1 (4.6)	20.8 (4.0)	0.79
**At Birth**	**Newborn weight (grams)**	3053.7 (549.6)	3133.4 (528.6)	2794.4 (552.8)	0.03
	**SBP (mmHg)**	120.0 (11.7)	120.2 (11.3)	119.3 (13.3)	0.79
	**DBP (mmHg)**	72.1 (9.6)	72.3 (9.2)	71.3 (10.9)	0.70
	**Maternal weight (Kg)**	83.5 (15.1)	82.7 (14.4)	86.2 (17.3)	0.43
	**Gestational age (weeks)**	38.1 (1.8)	38.3 (1.4)	37.6 (2.0)	0.11

BMI (body mass index), SBP (systolic blood pressure), DBP (diastolic blood pressure), LDL (low density lipoprotein), HDL (high density lipoprotein), HbA1c (Hemoglobin A1c), HOMA-IR (Homeostatic model assessment for insulin resistance), ALP (alkaline phosphatase), ALT (alanine transaminase) or AST (aspartate aminotransferase).

### Global Metabolic Differences Between Control and PCOS Participants at Second Trimester

**Figure 2:**
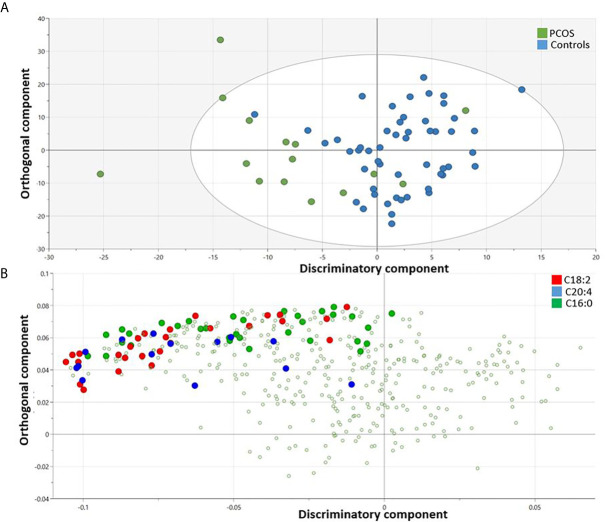
OPLS-DA model comparing metabolites from controls and PCOS mothers. Score plot shows one dimensional separation on the x-axis between controls and PCOS mothers **(A)** and the corresponding loading plot indicates triacylglycerols containing enriched fatty acids (C20:4, C18:2 and C16:0) that are responsible for the group separation **(B)**.

An OPLS-DA model comparing the metabolic profile of PCOS and control mothers indicated one class-discriminatory component and one orthogonal component, accounting for 40% of the variation in the study group Y-variable (PCOS groups). The Variable Importance in Projection (VIP) list indicated the discriminatory effects of triacylglycerols containing the following fatty acids (C20:4, C18:2 and C16:0) (**Supplementary Table S1**). The score plot exhibits an x-axis that separates the controls from PCOS group (**Figure 2A**) and the corresponding loading plot indicates triacylglycerols containing these fatty acids from the VIP list (C20:4, C18:2 and C16:0) (**Figure 2B**).

### Metabolites Differentiating Controls From PCOS Participants

**Figure 3:**
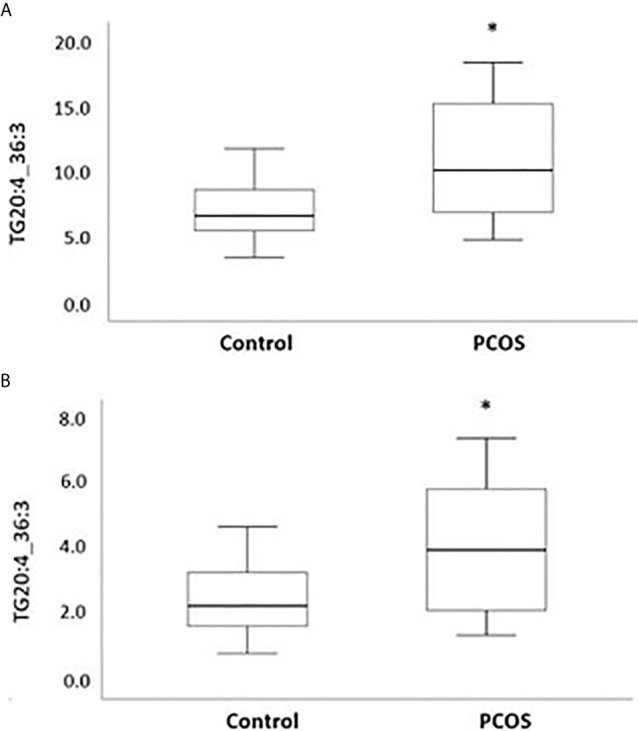
Boxplots representing metabolites increased with PCOS. Differences between controls and PCOS were tested by linear regression. Medians and interquartile range (arbitrary units) are shown for plasma levels of TG20.4_36.3 **(A)** and TG.18.2_38.6 **(B)**. (*) FDR level of significance of ≤ 0.05 was used.

Following the OPLS-DA multivariate analysis, univariate regression models were utilized to identify metabolites that differentiate PCOS from controls (**Table S3**). First analysis showed metabolites (mostly triacylglycerols) exhibiting significant differences between PCOS and controls at FDR (<0.1) level of significance (**Table 2**), with two (TG.20.4_36.3, TG.18.2_38.6) reaching FDR level of significance (<0.05) (**Figure 3**). A second analysis considering fatty acid composition of nominally significant triacylglycerols, revealed enrichment of triacylglycerols containing C20:4, C18:2 and C16:0 in the PCOS group (p=0.0003, <0.00001 and 0.03, respectively) (also highlighted in **Figure 2B**).

**Table 2:** Metabolites differentiating controls from PCOS women in their second trimester.

Metabolite	Sup-pathway	Super pathway	Estimate	SE	P value	FDR
TG.20.4_36.3	Triacylglycerols	Lipids	3.8	0.9	0.00007	0.031
TG.18.2_38.6	Triacylglycerols	Lipids	1.4	0.4	0.00020	0.047
TG.20.4_36.4	Triacylglycerols	Lipids	1.9	0.5	0.00036	0.052
TG.22.5_34.2	Triacylglycerols	Lipids	3.0	0.8	0.00067	0.052
TG.18.1_38.6	Triacylglycerols	Lipids	1.8	0.5	0.00083	0.052
TG.20.4_36.2	Triacylglycerols	Lipids	3.7	1.1	0.00087	0.052
TG.20.3_36.3	Triacylglycerols	Lipids	0.7	0.2	0.00109	0.052
TG.18.2_34.2	Triacylglycerols	Lipids	91.6	27.3	0.00133	0.052
DG.16.0_18.2	Diacylglycerols	Lipids	1.4	0.4	0.00136	0.052
DG.18.2_18.2	Diacylglycerols	Lipids	2.7	0.8	0.00167	0.052
TG.20.4_34.2	Triacylglycerols	Lipids	7.6	2.3	0.00169	0.052
TG.20.3_36.4	Triacylglycerols	Lipids	0.4	0.1	0.00176	0.052
TG.18.2_38.4	Triacylglycerols	Lipids	1.7	0.5	0.00182	0.052
TG.22.4_32.0	Triacylglycerols	Lipids	0.7	0.2	0.00183	0.052
TG.16.0_38.6	Triacylglycerols	Lipids	2.7	0.8	0.00187	0.052
Lactate	Carboxylic acids	Glycolysis	838.6	262.8	0.00218	0.052
TG.18.2_38.5	Triacylglycerols	Lipids	1.6	0.5	0.00222	0.052
TG.18.2_36.4	Triacylglycerols	Lipids	15.3	4.8	0.00226	0.052
TG.22.6_34.2	Triacylglycerols	Lipids	3.7	1.2	0.00227	0.052
AconAcid	Carboxylic acids	TCA cycle	0.3	0.1	0.00231	0.052
TG.16.0_40.8	Triacylglycerols	Lipids	0.9	0.3	0.00236	0.052
TG.22.5_32.0	Triacylglycerols	Lipids	1.4	0.4	0.00243	0.052
TG.16.0_38.5	Triacylglycerols	Lipids	4.4	1.4	0.00284	0.058
TG.18.2_36.3	Triacylglycerols	Lipids	30.8	10.1	0.00345	0.067
TG.16.0_40.7	Triacylglycerols	Lipids	1.7	0.6	0.00364	0.067
TG.22.4_34.2	Triacylglycerols	Lipids	1.2	0.4	0.00368	0.067
TG.22.6_32.0	Triacylglycerols	Lipids	1.6	0.5	0.00388	0.067
TG.20.2_34.2	Triacylglycerols	Lipids	1.6	0.5	0.00409	0.067
TG.18.0_38.6	Triacylglycerols	Lipids	0.4	0.1	0.00413	0.067
TG.22.5_34.1	Triacylglycerols	Lipids	2.9	1.0	0.00451	0.071
TG.18.2_32.0	Triacylglycerols	Lipids	33.5	11.4	0.00464	0.071
TG.20.4_32.0	Triacylglycerols	Lipids	3.5	1.2	0.00507	0.075
TG.16.0_40.6	Triacylglycerols	Lipids	1.8	0.6	0.00601	0.086
TG.16.0_36.4	Triacylglycerols	Lipids	49.8	17.7	0.00642	0.086
TG.18.1_36.4	Triacylglycerols	Lipids	18.3	6.5	0.00646	0.086
TG.20.3_34.2	Triacylglycerols	Lipids	2.2	0.8	0.00654	0.086
TG.20.4_34.1	Triacylglycerols	Lipids	7.3	2.6	0.00697	0.089
PC.aa.C36.4	Phosphatidylcholine	Lipids	50.1	18.0	0.00718	0.089
TG.17.0_36.4	Triacylglycerols	Lipids	0.6	0.2	0.00787	0.091
TG.20.4_34.3	Triacylglycerols	Lipids	0.8	0.3	0.00787	0.091
TG.22.6_34.1	Triacylglycerols	Lipids	4.2	1.5	0.00792	0.091
TG.20.4_34.0	Triacylglycerols	Lipids	1.1	0.4	0.00847	0.095
TG.20.3_32.0	Triacylglycerols	Lipids	1.2	0.5	0.00908	0.100

Linear regression was performed to identify significant metabolites differentiating Controls from PCOS using the R statistical package after correcting for age, BMI and principle components (PC1 and PC2). Estimate (beta value), SE (standard error), FDR (False Discovery Rate).

### Indicative Biomarkers of Birth Weight

**Figure 4:**
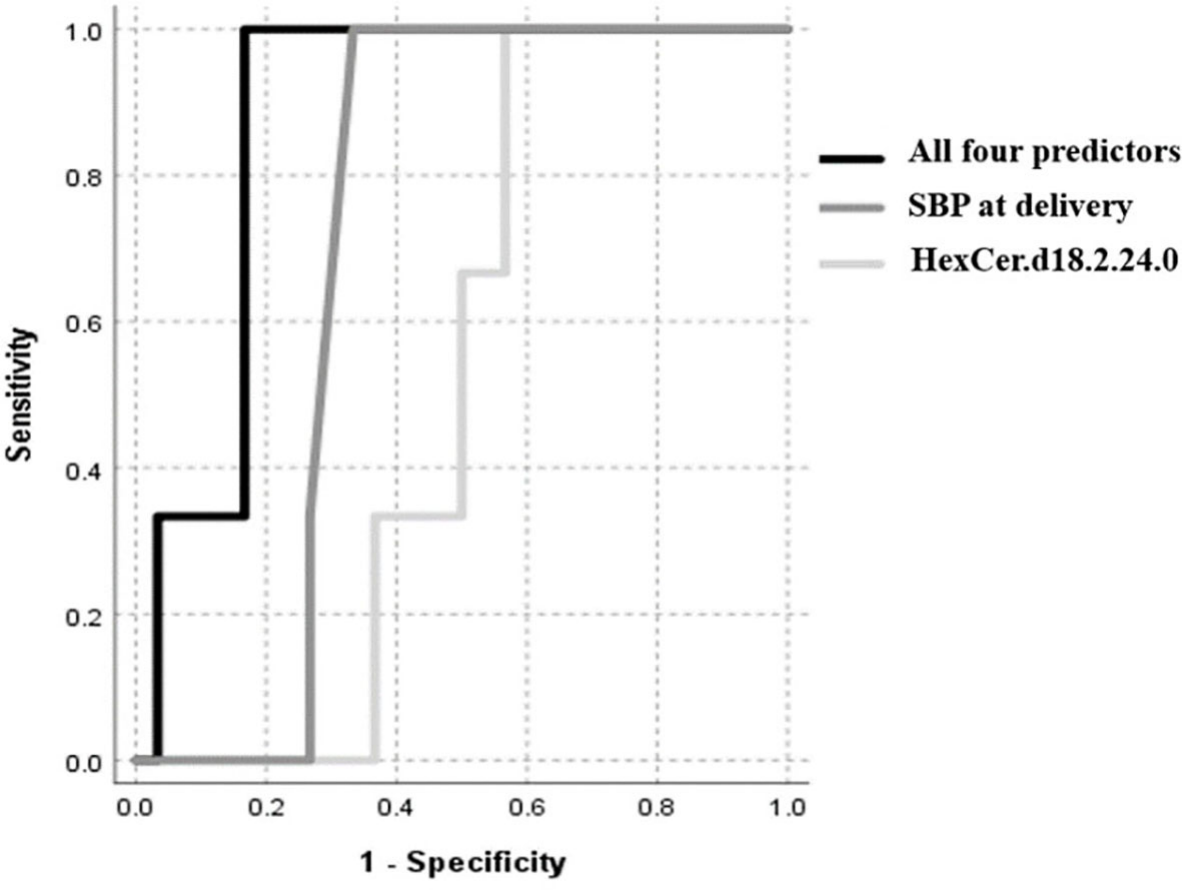
ROC curve of low birth weight (≤2500 grams), including maternal SBP at delivery, HexCer.d18.2.24.0, Cer.d18.0.24.1 and serine.

An automatic linear regression model was utilized to identify best predictors of birth weight regardless of study groups. The model revealed 4 indicators, three of which were lipids, that varied in their importance and direction (positive and negative) in indicating the birth weight (**Table 3**) with a combined AUC for all indicators of low birth weight (≤2500 grams, n=8) of 0.88 (95%CI: 0.75-1.005, p<0.001) (**Figure 4**).

**Table 3:** List of indicators associated with birth weight.

Predictor	Importance	Beta	P value	AUC(≤2500g)	Adjusted R^2^
SBP at delivery	0.22	-0.49	0.003	0.7	0.44
HexCer.d18.2.24.0.	0.14	-0.36	0.02	0.6	
Cer.d18.0.24.1.	0.08	-0.28	0.05	0.6	
Serine	0.06	0.51	0.001	0.4	

## Discussion

In the present study, we identified a distinct metabolic signature differentiating PCOS women from age, BMI and gestational age-matched controls, and revealed potential indicative metabolic biomarkers for the low birth weight of babies associated with PCOS. Our novel data indicate significant elevation in various serum triglycerides, particularly the polyunsaturated triglycerides C20:4_C34:3 and C18:2_C38:6, in the PCOS pregnant women. Although, triacylglycerols are not metabolically active, their breakdown products, including fatty acids and diacylglycerols, play important roles in cell signaling (8, 20). Indeed, our subsequent enrichment analysis showed significant elevations in triglycerides containing essential polyunsaturated fatty acid (PUFA) with critical cell signaling properties, namely C20:4 (arachidonic acid), C18:0 (linoleic acid) and C16:0 (palmitic acid) in the PCOS group. Several mass spectrometry-based studies have shown alterations in certain free fatty acids in the serum of PCOS women, including palmitoleic and oleic acids (21, 22). Furthermore, increased serum levels of linoleic acid were reported in PCOS obese patients (21), whereas elevated oleic acid and stearic acid levels were associated with the developmental competence of oocytes, which could contribute to lower pregnancies in PCOS women (21). These PUFAs were previously reported to play important roles in reproductive performance (23–25). Studies have shown that serum levels of arachidonic acid, linoleic acid, and docosahexaenoic acid were decreased in obese PCOS women compared to lean controls (14). This data highlights the important roles of these bioactive lipids in obesity-associated PCOS and PCOS in pregnant women. Previous studies have indicated that free fatty acids represent essential molecular mediators of various metabolic diseases including PCOS as they play a critical role in lipid metabolism with significant impact on cell growth, differentiation and metabolism (14). Oocyte fatty acid composition and their levels in the microenvironment could influence embryogenesis and pregnancy outcome (26, 27), by triggering endoplasmic reticulum stress and influencing oocyte maturation (21).

Our study has also revealed a 10% lower birth weight of babies in PCOS women compared to their matching controls: low birth weight is associated with a range of poor fetal outcomes including increased risk of neonatal death (28). Our data is in accord with previous studies that have reported an increased prevalence of small-for-gestational-age offspring in women with PCOS (5, 29) with no difference in gestational age at delivery. It is recognized that mothers with higher blood pressure had offspring with lower birth weight (30, 31) that we also showed here; however, in addition we report the novel finding of a negative correlation between maternal blood pressure in the second trimester and baby birth weight at delivery that may suggest a functional relationship between baby birth weight and maternal blood pressure as early as the second trimester. When considering potential indicative metabolic biomarkers of birth weight, our data revealed that SBP at delivery was the strongest predictor of low birth weight. Combined with the sphingolipids including the hexosylceramide HexCer (d18:2/24:0), the dihydroceramides Cer (d18:0/24:1) as well as the amino acid serine, these 4 markers best indicated low birth weight in our study.

The underlying mechanism of increased maternal blood pressure and associated lower birth weight is not well characterized. Elevated maternal blood pressure could potentially reflect a compensatory mechanism for placental dysfunction and a consequence of restricted fetal growth (32). The relationship between the two identified ceramides (HexCer.d18.2.24.0 and Cer.d18.0.24) and low birthweight could be reflecting their association with maternal insulin resistance, as our data has shown, causing lower gestational weight. Elevated levels of hexCer were previously shown to be associated with insulin resistance its driven lipotoxicity (33). Previous studies in rats have indicated that high muscle stearoyl- and oleoyl-ceramide content is associated with increased insulin resistance (34). Increased serine levels with lower gestational age could reflect the close correlation between serine levels and IR and obesity. Increased glycolysis may represent the key factor for increased serine serum levels in PCOS patients (35). Whether serine is an independent predictor of low birth weight, or simply an indirect marker for glucose intolerance, abnormal glucose metabolism and insulin resistance, it remains to be investigated. Serine phosphorylation hypothesis may provide a common biological mechanism for the two principal features of PCOS hyperandrogenemia and insulin resistance, however it t remains to be proven (36), however it remains to be verified. Further studies are warranted to characterize the functional relevance and the predictive value of the identified indicators of low birth weight at second trimester for diagnostic and potentially therapeutic interventions.

### Study Limitations

Despite the relative low number of participants in the PCOS group, the metabolic differences between PCOS and matching controls were large enough to be detected following multiple testing correction. However, larger studies are warranted to rule out the possibility of false negatives due to low study power. Furthermore, the cross-sectional design may have reduced the ability to assess the metabolic changes during pregnancy and the interpretation of data from a pathophysiological angle. Additionally, the observational nature of the study warrants functional validation before inferring any functionality. Other unmeasured factors were not accounted for such as dietary habits, medication and other unknown environmental factors; however, correcting for principle components may have partially accounted for some of these confounders. Larger studies with dynamic measurement of metabolites during pregnancy in different matrixes, combined with functional validation, would certainly improve our understanding of the metabolic signature of PCOS and its impact on pregnancy outcome.

In conclusion, these data indicate differences in the metabolic signature between PCOS and control pregnant women at second trimester, and highlight the indicative potential of triacylglycerols as metabolic markers of low birth weight. This data needs further validation to improve our understanding of the pathophysiology of PCOS in pregnancy and its impact on pregnancy outcome.

## Data Availability Statement

The datasets used and/or analyzed during the current study are available from the corresponding author on reasonable request.

## Ethics Statement

The studies involving human participants were reviewed and approved by Institutional Review Boards (IRBs) of the Hamad Medical Corporation (15101/15) and Weill Cornell Medicine in Qatar (15-00016). The patients/participants provided their written informed consent to participate in this study.

## Author Contributions

ID carried out the statistical analysis and wrote the paper. YM, MR, OB, LA, AB, and AA-S helped with data analysis and reviewing the paper. MB and SA were involved in study design, sample collection and data analysis. ME and NM were lead principle investigators, designed the experiments, supervised progress, analyzed data and wrote and approved the final version of the article. ME and NM are responsible for the integrity of the work as a whole. All authors contributed to the article and approved the submitted version.

## Funding

This research was sponsored by QNRF, Grant no. NPRP10-1205-160010 (NM) and NPRP13S-1230-190008 (ME and NM).

## Conflict of Interest

The authors declare that the research was conducted in the absence of any commercial or financial relationships that could be construed as a potential conflict of interest.

The handling editor declared a past co-authorship with one of the authors MR.
